# YY1-induced lncRNA ZFPM2-AS1 facilitates cell proliferation and invasion in small cell lung cancer via upregulating of TRAF4

**DOI:** 10.1186/s12935-020-1157-7

**Published:** 2020-04-03

**Authors:** Zhijun Yan, Qilian Yang, Min Xue, Sheng Wang, Weijun Hong, Xiwen Gao

**Affiliations:** 1 Department of Respiratory Medicine, Minhang Hospital, Fudan University, 170 Xinsong Road, Shanghai, 201199 China; 2 Department of Pharmacy, Minhang Hospital, Fudan University, 170 Xinsong Road, Shanghai, 201199 China; 3 Institutes of Biomedical Sciences, Fudan University, 131 Dongan Road, Shanghai, 200032 China

**Keywords:** ZFPM2-AS1, miR-3612, TRAF4, YY1, SCLC

## Abstract

**Background:**

Newly identified lncRNA zinc finger protein, FOG family member 2 antisense RNA 1 (ZFPM2-AS1) is identified as an oncogenic gene. However, the role of ZFPM2-AS1 in small cell lung cancer (SCLC) is poorly comprehended.

**Methods:**

The expression of genes in SCLC tissues and cells was measured by qRT-PCR. Colony formation, EdU, CCK-8, transwell and wound healing as well as in vivo assays revealed the function of ZFPM2-AS1 in SCLC. ChIP, luciferase reporter, RIP and RNA pull down assays demonstrated the binding relation among genes.

**Results:**

ZFPM2-AS1 was significantly upregulated in SCLC tissues and cells. ZFPM2-AS1 deficiency attenuated SCLC cell proliferation, invasion and migration. In addition, ZFPM2-AS1 was transcriptionally activated by Yin Yang 1 (YY1) factor. Further, miR-3612 was confirmed as downstream miRNA of ZFPM2-AS1. Moreover, TNF receptor associated factor 4 (TRAF4) was the target gene of miR-3612 in SCLC. ZFPM2-AS1, miR-3612 and TRAF4 jointly constituted a competing endogenous RNA (ceRNA) network in SCLC. Finally, TRAF4 could countervail ZFPM2-AS1 downregulation-mediated function on SCLC cell proliferation and invasion in vitro and tumor growth in vivo.

**Conclusion:**

Our study elucidated the oncogenic effect of ZFPM2-AS1 in SCLC progression, indicating it may be a therapeutic target for SCLC.

## Background

Statistically, lung cancer is characterized with the highest incidence, occupying 11.6% of cancer-induced mortalities [1]. Domestically, the estimated number of diagnosed lung cancer was 733,300 with 610,200 death cases in 2015, indicating the life-threatening influence of lung cancer [2]. Compared with non-small‐cell lung cancer (NSCLC), small‐cell lung cancer (SCLC) only accounts for a minor part in lung cancer. More surprisingly, SCLC is substantiated as the subtype with highest risk of aggressiveness due to its rapid growth and early invasiveness [3, 4]. Additionally, 5-year survival of SCLC is lower than 10%, meanwhile, the dreadful median survival of it is unsatisfactory [5–7]. Nevertheless, the number of existing research focusing on SCLC is limited.

The transcribed RNA contains non-coding RNAs (ncRNAs) in large quantity, which are limited in protein-coding [8]. Long non-coding ncRNAs (lncRNAs), containing over 200 nucleotides in length, belong to ncRNAs. Further lncRNAs are validated to exert multiple functions in biological processes [9]. Their epigenetic silencing was constituted by lncRNA-miRNA interplay and lncRNA-protein interaction [10]. MicroRNAs (miRNAs), typically 18–200 nucleotides in length, has extensively reported by researches in tumor, and up to 30% of mRNAs are regulated by thousands of miRNAs [9]. LncRNAs could competitively bind miRNAs to modulate the expression of target mRNAs, forming a ceRNA network [11, 12]. For instance, lncRNA PVT1 enhances insulin like growth factor 1 receptor (IGF1R) expression to promote cell proliferation and invasion in papillary thyroid carcinoma by acting as ceRNA of miRNA-30a [13]. LncRNA RP4 serves as a ceRNA for SH3GLB1 to sponge miR-7-5p in colorectal cancer [14]. LINC00488 upregulates TLN1 as a ceRNA in hepatocellular carcinoma cell growth through sponging miR-330-5 [15]. Above all, lncRNAs could affect tumorigenesis via gene modulation. Moreover, various cancers including SCLC are functionally associated with abnormally expressed lncRNAs [16, 17]. For example, highly expressed AFAP1-AS1 is correlated with nasopharyngeal carcinoma metastasis [18]. And overexpressed LINC00511 is implicated in breast cancer tumourigenesis and stemness [19]. Nevertheless, the investigation about participation of lncRNAs in SCLC is limited.

ZFPM2-AS1 interested us due to its close association with diverse cancers. In previous researches, ZFPM2-AS1 facilitated gastric cancer through inhibiting the p53 pathway [20]. Besides, it served as prognosis biomarker in hepatocellular carcinoma [21]. Moreover, ZFPM2-AS1 induced renal cell cancer tumorigenesis through targeting miR-137 [22]. Also, ZFPM2-AS1 regulated miR-18b-5p/VMA21 axis to promote lung adenocarcinoma progression [23]. Therefore, this study aimed to investigate the role and regulatory mechanism of ZFPM2-AS1 in SCLC.

## Materials and methods

### Patient tissues

In this study, total 54 pairs of small cell lung cancer (SCLC) tissues and matched normal tissues were collected under the approval from the Ethics Committee of Minhang Hospital, Fudan University. All participants were not treated with radiotherapy and chemotherapy before operation. Tumor tissues and respective normal tissues were snap-frozen in liquid nitrogen at − 80 °C until requested.

### Cell culture

Human bronchial epithelial cell (16-HBE) and human SCLC cells (DMS-53, H446, SHP-77, H69) were obtained from American Type Culture Collection (ATCC, Rockville, MD, USA). Cells were cultured in DMEM (Thermo Fisher Scientific, Waltham, MA, USA) with 10% fetal bovine serum (FBS, Gibco, Carlsbad, CA, USA) and 1% penicillin or streptomycin (Gibco) at 37 °C with 5% CO_2_.

### Transfection

The short hairpin RNAs (shRNAs) targeting ZFPM2-AS1 (sh-ZFPM2-AS1#1/#2/#3), YY1 (sh-YY1#1/#2) and negative control (shNC) were obtained from Genechem (Shanghai China), transfected into DMS-53 and SHP-77 cells, respectively. The pcDNA3.1/YY1, pcDNA3.1/ZFPM2-AS1, pcDNA3.1/TRAF4 and empty pcDNA3.1 vector were constructed by KeygenBiotech (Nanjing, China), along with miR-3612 mimics/inhibitor and NC mimics/inhibitor. Using Lipofectamine 2000 (Invitrogen, Carlsbad, Calif, USA), cell transfection was conducted.

### Quantitative real-time polymerase chain reaction (qRT-PCR)

TRIzol reagent (Invitrogen, Carlsbad, CA, USA) was applied to extract total RNA from cells. Then, Reverse Transcription Kit was used to conduct the reverse transcription between total RNA and cDNA. qRT-PCR was performed in line with SYBR-Green Real-Time PCR Kit (Takara, Tokyo, Japan). Relative gene expression was operated with 2^−ΔΔCt^ method and normalized to the GAPDH or U6.

### Colony formation assay

DMS-53 or SHP-77 cells were cultivated in 6-well plates after transfection. Two weeks later, cells were washed with PBS (Solibao technology, Beijing, China), fixed in methanol (Solibao technology) and stained with crystal violet (Solibao technology). Finally, the colony numbers were counted manually.

### Cell proliferation assay

The viability of DMS-53 or SHP-77 cells was detected by the use of CCK-8 Kit (Sigma-Aldrich, Carlsbad, USA). Cells were placed into 96-well plates at specific time points. 10 μl CCK-8 were added into each well for 4 h. To measure the absorbance at 450 nm, the microplate reader (Bio-Rad, Hercules, CA, USA) was utilized.

### Transwell assay

2 × 10^4^ cells were re-suspended in serum-free medium and were cultured in the top transwell chamber (BD Biosciences, NY, USA). Then, 10% PBS was added to the lower chamber. Migrated cells were fixed with methanol and dyed in crystal violet, followed by observation under microscope (Leica Microsystems, Wetzlar, Germany). Invasion assay was conducted the same as above, but pre-coated with matrigel (BD Bioscience).

### EdU incorporation assay

Based upon the standardized guides, cells were cultured with DMEM supplemented with 300 μl EdU for 2 h after transfection. Then, cells were fixed with 4% formaldehyde (Solibao technology) at room temperature for 30 min. After staining, cells were observed under fluorescence microscope (Leica Microsystems). Besides, EdU-positive cells (stained in red) are cells that were proliferating, and DAPI stained cells (stained in blue) were total cells.

### Wound healing assay

Cells were seeded to 6-well plates and cultivated with general cell growth fluid. The sterile pipette was used to make scratches in cell layers when cells were paved. Once cells were cleaned, the medium was replaced. Ultimately, the images were collected and analyzed.

### Western blot

The cells were lysed with RIPA lysis buffer containing protease inhibitor and the complete DNA was obtained. After detecting the protein concentrations by BCA method, proteins were isolated by sodium dodecyl sulfate polyacrylamide gel electrophoresis (SDS-PAGE) gel electrophoresis, and were then transferred to PVDF. The membrane was incubated with primary antibodies overnight after PVDF was sealed with skim milk. The primary antibodies including: anti-YY1 (ab109237, Abcam, Cambridge, USA), anti-CBX5 (ab109028, Abcam) anti-TRAF4 (ab190986, Abcam), anti-TAOK1 (ab197891, Abcam) and anti-GAPDH (ab8245, Abcam) at 4 °C. Following that, the chemiluminescence system (Hubei Biossci Biotechnology, Wuhan, China) was employed for detecting the protein.

### Chromatin immunoprecipitation (ChIP) assay

ChIP assay was implemented with the application of EZ ChIP™ Chromatin Immunoprecipitation Kit for cells (Millipore, Bedford, MA), following then standard method. DMS-53 or SHP-77 cells were fixed with 1% formaldehyde for 15 min’s crosslink and lysed, and then the DNA was sonicated for shearing DNA into 500-bp fragments. DNA samples were precipitated with anti-IgG or anti-YY1 antibody for 6 h, in the presence of 30 μl of magnetic beads. Subsequently, the precipitated chromatin DNA was collected, extracted and purified for qRT-PCR analysis.

### Luciferase reporter assay

The ZFPM2-AS1 promoter was sub-cloned into pGL3 vector to construct reporter plasmids and then was co-transfected with pcDNA3.1/YY1 or sh-YY1 in DMS-53 and SHP-77 cells. ZFPM2-AS1 or TRAF4 fragments containing the predicted binding site of miR-3612 were sub-cloned into pmirGLO dual-luciferase plasmid (Promega, Madison, WI, USA) and named as ZFPM2-AS1-WT/TRAF4-WT. ZFPM2-AS1-MUT/TRAF4-MUT was generated by mutations in specific binding site. SHP-77 or DMS-53 cells were co-transfected with indicated plasmids or miR-3612 mimics, NC mimics, miR-3612 inhibitor or NC inhibitor. After 48 h, the dual-luciferase activity was assessed by the Dual-Luciferase Reporter Assay System (Beinuo Biotechnology, Shanghai, China).

### Subcellular fractionation

According to the indicated protocols, Cytoplasmic and Nuclear RNA Purification Kit (Norgen, Ontario, Canada) was employed for isolating and purifying cytoplasmic and nuclear RNA. Expression levels of ZFPM2-AS1 were assessed by qRT-PCR analysis. GAPDH and U1 were used to control of cytoplasm or nucleus.

### RNA pull down assay

Bio-miR-miR-3612-Mut/Wt and Bio-NC were acquired via biotin-labeling miR–miR-3612-Wt/Mut and NC. ZFPM2-AS1 biotin probe and no ZFPM2-AS1 probe constructed by GenePharma were treated with M-280 Streptavidin magnetic beads (Invitrogen). Then, cells were collected, lysed, and incubated with probe-coated beads overnight for 48 h. RNA complexes after purification were detected by qRT-PCR assay.

### RNA immunoprecipitation (RIP) assay

Cells were lysed, incubated with anti-Ago2 and anti-IgG antibodies (Millipore, Bellerica, MA, USA) coated on magnetic beads in RIP buffer. The precipitated RNAs were isolated for qRT-PCR.

### Fluorescence in situ hybridization (FISH) assay

ZFPM2‐AS1 subcellular localization was detected using a FISH Kit (Roche, Basel, Switzerland). Cells were mixed with paraformaldehyde for fixation. Then, ZFPM2‐AS1 probe (Sigma-Aldrich) hybridization solution was added. Cell nucleus was stained for 10 min at room temperature. Lastly, the laser confocal scanning microscopy (FV1000; Olympus, Tokyo, Japan) was used.

### Animal study

Female BALB/C athymic nude mice (18–20 g; 6-week-old) were procured from the National Laboratory Animal Center (Beijing, China) and kept under the SPF-grade animal lab. The animal study was undertaken with the approval from the Animal Research Ethics Committee of Minhang Hospital, Fudan University. In vivo study was implemented via subcutaneous injection of 5 × 10^6^ transfected SCLC cells to mice for 28 days, with tumor volume monitored every 4 days. The tumors collected from killed mice were weighed for analysis.

### Statistical analysis

GraphPad Prism 7 software package (Graph-Pad Software, Inc., La Jolla, CA, USA) was used for the statistical analysis. Quantitative data were showed as mean ± standard deviation. Student’s-test and ANOVA was used to compare the difference of groups. P < 0.05 had statistically significance. The experiment was conducted at least in thrice.

## Results

### Down-regulation of ZFPM2-AS1 suppressed malignant behaviors of SCLC cells

**Fig. 1:**
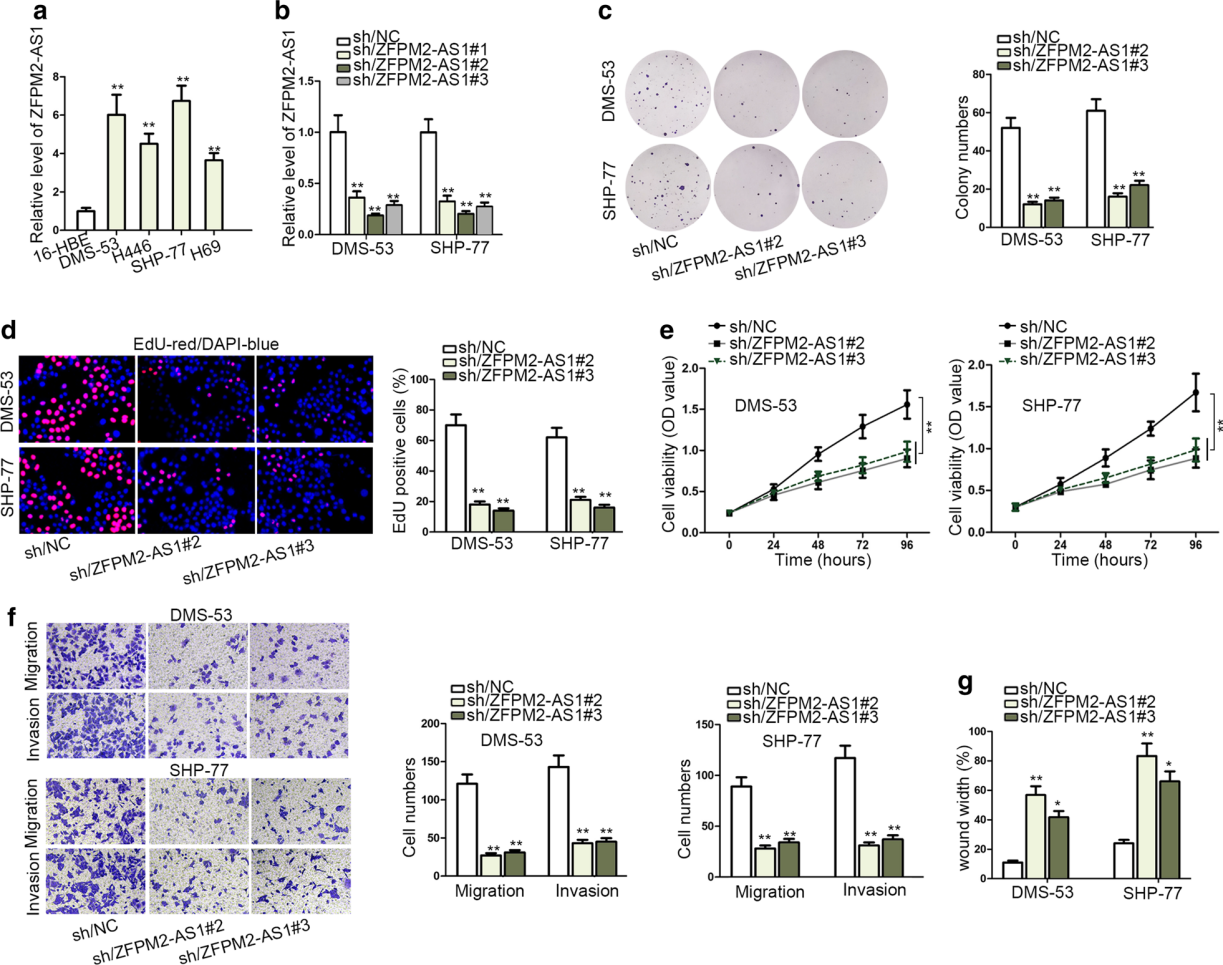
Downregulation of ZFPM2-AS1 suppressed malignant behaviors of SCLC cells. **a** qRT-PCR results of ZFPM2-AS1 level in SCLC cell lines and controls (16-HBE). **b** qRT-PCR results of ZFPM2-AS1 level following treating DMS-53 and SHP-77 cells with shRNAs targeting ZFPM2-AS1. **c**, **d** Colony formation and EdU analyses of DMS-53 and SHP-77 cell proliferation in response to ZFPM2-AS1 knockdown. In EdU assay, EdU-positive cells (stained in red) are cells that were proliferating, and DAPI stained cells (stained in blue) were total cells. **e** CCK-8 evaluated DMS-53 and SHP-77 cell viability when downregulating ZFPM2-AS1. **f** Transwell assay detected DMS-53 and SHP-77 cell invasion and migration after silencing ZFPM2-AS1. **g** Wound healing assay assessed DMS-53 and SHP-77 cell migration upon ZFPM2-AS1 silencing. *P < 0.05, **P < 0.01

Firstly, we detected that ZFPM2-AS1 was upregulated in SCLC tissues compared with matched normal group (Additional file 1: Fig. S1A). Then the expression pattern of ZFPM2-AS1 in SCLC cells (H69, DMS-53, H446, SHP-77) and human bronchial epithelial cells (16-HBE) was measured. Results of qRT-PCR indicated that ZFPM2-AS1 was highly expressed in SCLC cell lines rather control cells. Besides, DMS-53 and SHP-77 cells contained higher level of ZFPM2-AS1 than other two SCLC cells (Fig. 1a). ZFPM2-AS1 was knocked down via transfecting sh/ZFPM2-AS1#1/2/3 plasmids into DMS-53 and SHP-77 cells. And knockdown efficiency of sh/ZFPM2-AS1#/2/3 was more obvious (Fig. 1b). Therefore, loss-of-function assays of ZFPM2-AS1 were performed utilizing sh/ZFPM2-AS1#2 and sh/ZFPM2-AS1#3 plasmids. ZFPM2-AS1 inhibition strikingly decreased SCLC cell proliferation, evidenced by colony formation and EdU assays (Fig. 1c, d). CCK-8 assay further verified this result. Cell viability was impaired by ZFPM2-AS1 downregulation (Fig. 1e). Following ZFPM2-AS1 depletion, the migration and invasion of cells were slowed down (Fig. 1f). Meanwhile, wound healing assay convinced that silencing of ZFPM2-AS1 would weaken cell migration ability (Fig. 1g, Additional file 1: Figure S1B). To sum up, SCLC cell proliferation, invasion and migration were depressed by knockdown of ZFPM2-AS1, which was upregulated in SCLC tissues and cells.

### ZFPM2-AS1 was transcriptionally activated by YY1

**Fig. 2:**
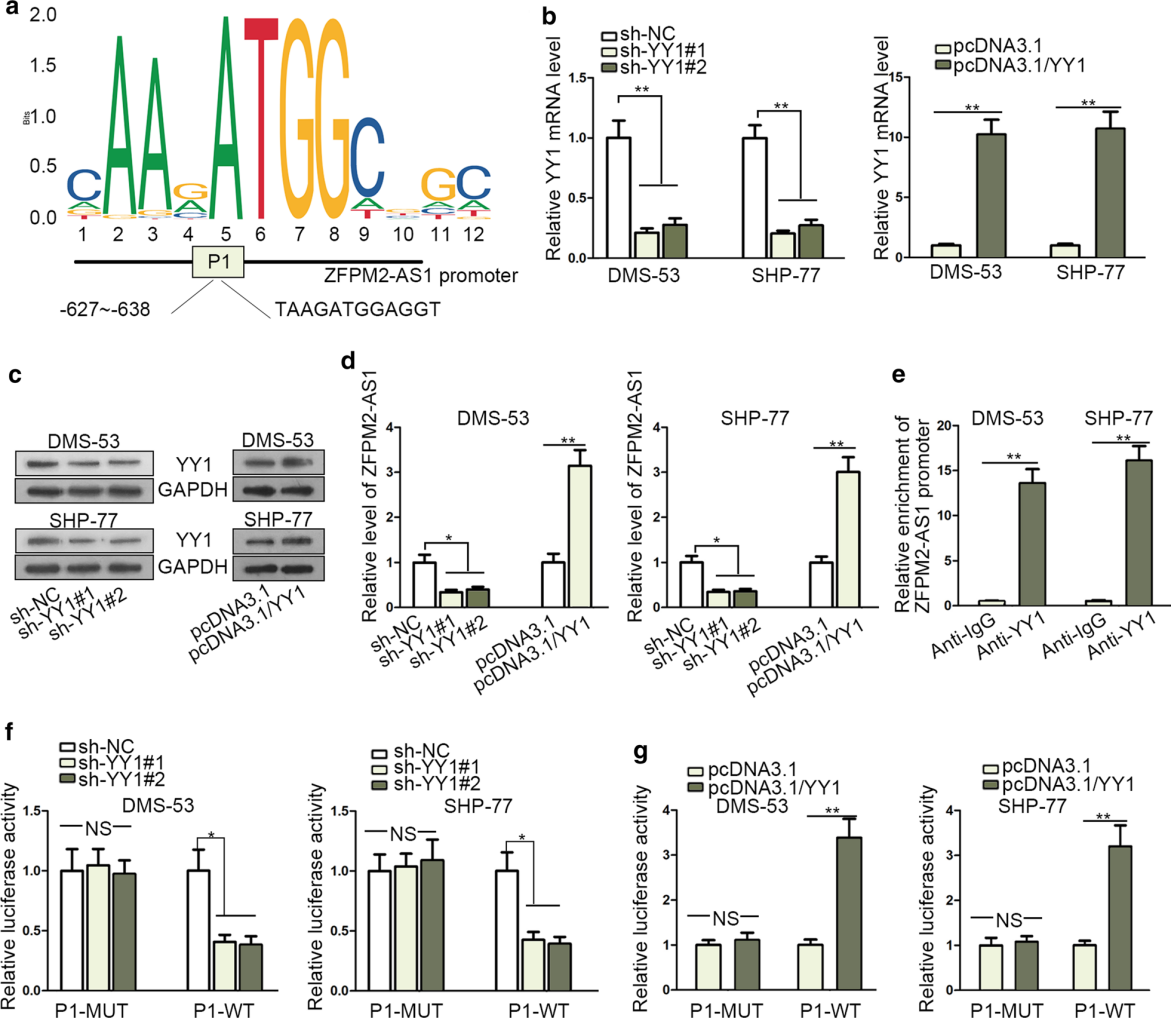
ZFPM2-AS1 was transcriptionally activated by YY1. **a** YY1 binding motif and the related binding site for YY1 in ZFPM2-AS1 promoter were acquired via searching JASPAR. **b**, **c** qRT-PCR and western blot analyses of YY1 mRNA level upon YY1 elevation or suppression. **d** The expression of ZFPM2-AS1 in response to YY1 elevation or suppression was detected by qRT-PCR. **e** The binding relation between YY1 and ZFPM2-AS1 promoter was ensured by ChIP using anti-YY1. **f**, **g** The luciferase activity of wild ZFPM2-AS1 promoter (P1) in differently transfected groups was detected by luciferase reporter assay. *P < 0.05, **P < 0.01. NS: no significance

The implication of transcription factor in lncRNAs couldn’t be ignored [24]. Therefore, the possible upstream factors of ZFPM2-AS1 were analyzed via searching UCSC (http://genome.ucsc.edu/). Data indicated that YY1 was qualified one. Further, through JASPAR (http://jaspar.genereg.net/), the binding site YY1 between and ZFPM2-AS1 promoter (P1) was predicted (Fig. 2a). In addition, the upregulated expression tendency of YY1 in SCLC tissues was verified by qRT-PCR (Additional file 1: Fig. S1C). Next, the regulatory role of YY1 on ZFPM2-AS1 expression was detected. The satisfactory efficiency of YY1 overexpression or knockdown was confirmed by qRT-PCR and western blot (Fig. 2b, c). Subsequently, ZFPM2-AS1 expression was decreased or increased when downregulating or upregulating YY1 (Fig. 2d). Moreover, the binding relation between YY1 and ZFPM2-AS1 promoter was well illuminated in ChIP assay. Abundant enrichment of ZFPM2-AS1 promoter was observed in anti-YY1 group (Fig. 2e). Moreover, luciferase reporter assay detected that luciferase activity of wild ZFPM2-AS1 promoter (P1) was reduced upon YY1 knockdown while that of mutant ZFPM2-AS1 (P1) presented no change. On the contrary, upregulation of YY1 led to an increase in luciferase activity of wild ZFPM2-AS1 promoter (Fig. 2f, g). In a summary, ZFPM2-AS1 was transcriptionally activated by transcription factor YY1 in SCLC.

### ZFPM2-AS1 acted as sponge of miR-3612

**Fig. 3:**
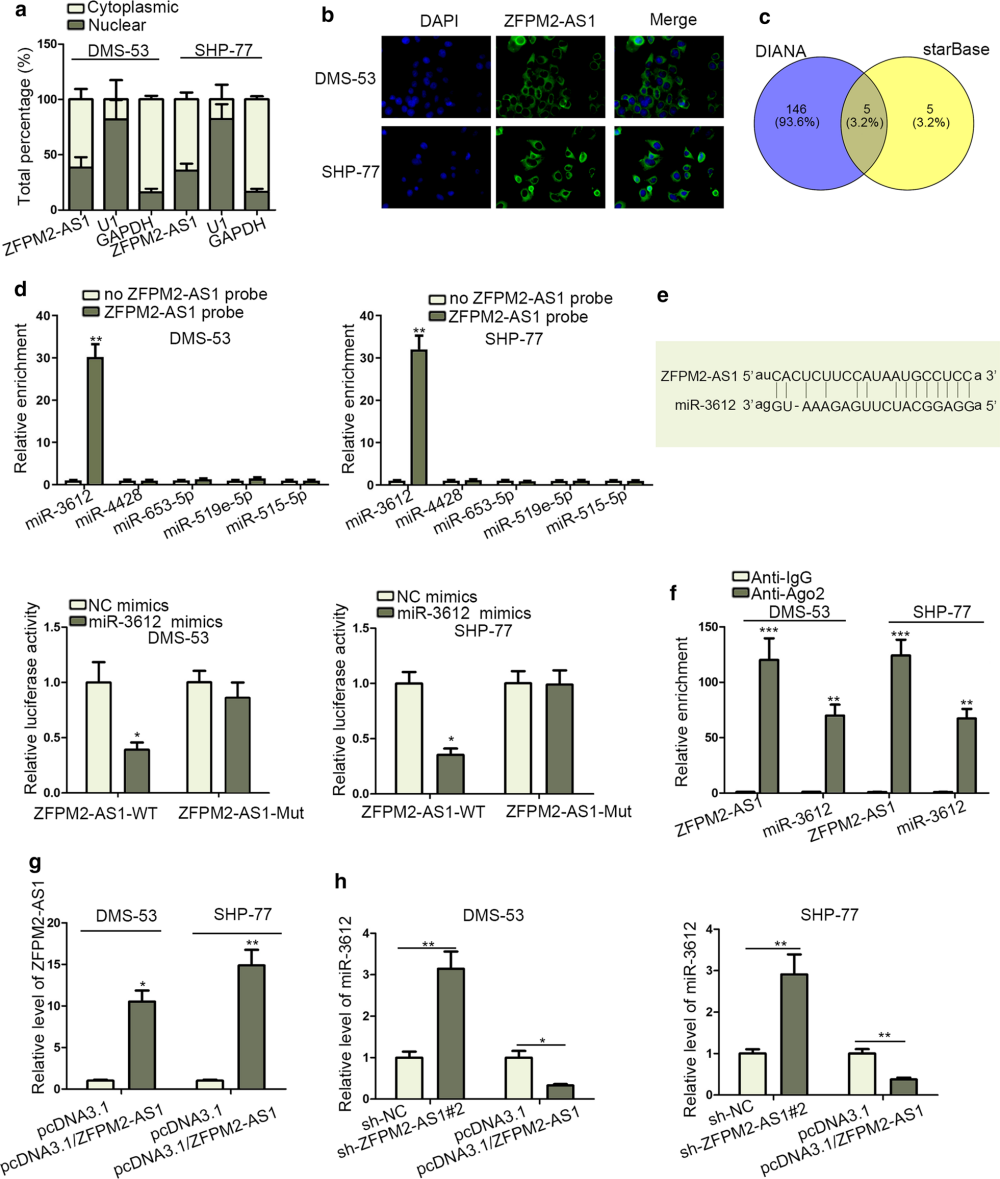
ZFPM2-AS1 acted as sponge of miR-3612. **a** Both cytoplasmic and nuclear expression of ZFPM2-AS1 was measured via qRT-PCR after subcellular fractionation. **b** FISH probed the expression of ZFPM2-AS1 in cytoplasm and nucleus. **c** DIANA and starBase revealed the predicted miRNAs that might bind with ZFPM2-AS1. **d** RNA pull-down assay detected the enrichment of the indicated miRNAs in ZFPM2-AS1 probe group. **e** The binding site between miR-3612 and ZFPM2-AS1 was exhibited (upper). Luciferase activity of ZFPM2-AS1-WT or ZFPM2-AS1-MUT was monitored by luciferase reporter assay following NC mimics or miR-3612 mimics transfection (lower). **f** RIP assay examined the enrichment of ZFPM2-AS1 and miR-3612 in Anti-Ago2 group. **g** Overexpression efficiency of ZFPM2-AS1 in SHP-77 and DMS-53 cells was detected by qRT-PCR. **h** qRT-PCR evaluated the regulatory effects of ZFPM2-AS1 on miR-3612 expression in SHP-77 and DMS-53 cells. *P < 0.05, **P < 0.01, *** P < 0.001

The downstream regulation mechanism of ZFPM2-AS1 was then explored. Firstly, subcellular fractionation assay determined that ZFPM2-AS1 expression mainly distributed in SCLC cytoplasm, further was confirmed by FISH assay (Fig. 3a, b). Then five miRNAs (miR-515-5p, miR-519e-5p, miR-653-5p, miR-4428, miR-3612) were discovered to possibly bind with ZFPM2-AS1 by starBase (http://starbase.sysu.edu.cn/) and DIANA (http://carolina.imis.athena-innovation.gr/diana_tools/web/index.php?r=site%2Ftools) (Fig. 3c). RNA pull-down assay was used to screen out miR-3612 due to its obvious enrichment in ZFPM2-AS1 probe-precipitated complex compared with other four miRNAs (Fig. 3d). Besides, miR-3612 was downregulated in SCLC tissues in comparison with control group (Additional file 1: Fig. S1D). As shown in Fig. 3e, the binding site between ZFPM2-AS1 and miR-3612 were predicted by starBase. Subsequently, luciferase reporter assay examined that miR-3612 mimics efficiently decreased the luciferase activity of ZFPM2-AS1-WT in SHP-77 and DMS-53 cells. In addition, large enrichments of ZFPM2-AS1 and miR-3612 were measured in anti-Ago2 group (Fig. 3f). Next, the regulation role of ZFPM2-AS1 in miR-3612 level was assessed. ZFPM2-AS1 expression was increased in pcDNA3.1/ZFPM2-AS1 transected cells (Fig. 3g). MiR-3612 level was negatively regulated by ZFPM2-AS1 in SHP-77 and DMS-53 cells (Fig. 3h). In conclusion, miR-3612 was sponged by ZFPM2-AS1 in SCLC cells.

### ZFPM2-AS1 upregulated TRAF4 expression via sequestering miR-3612

**Fig. 4:**
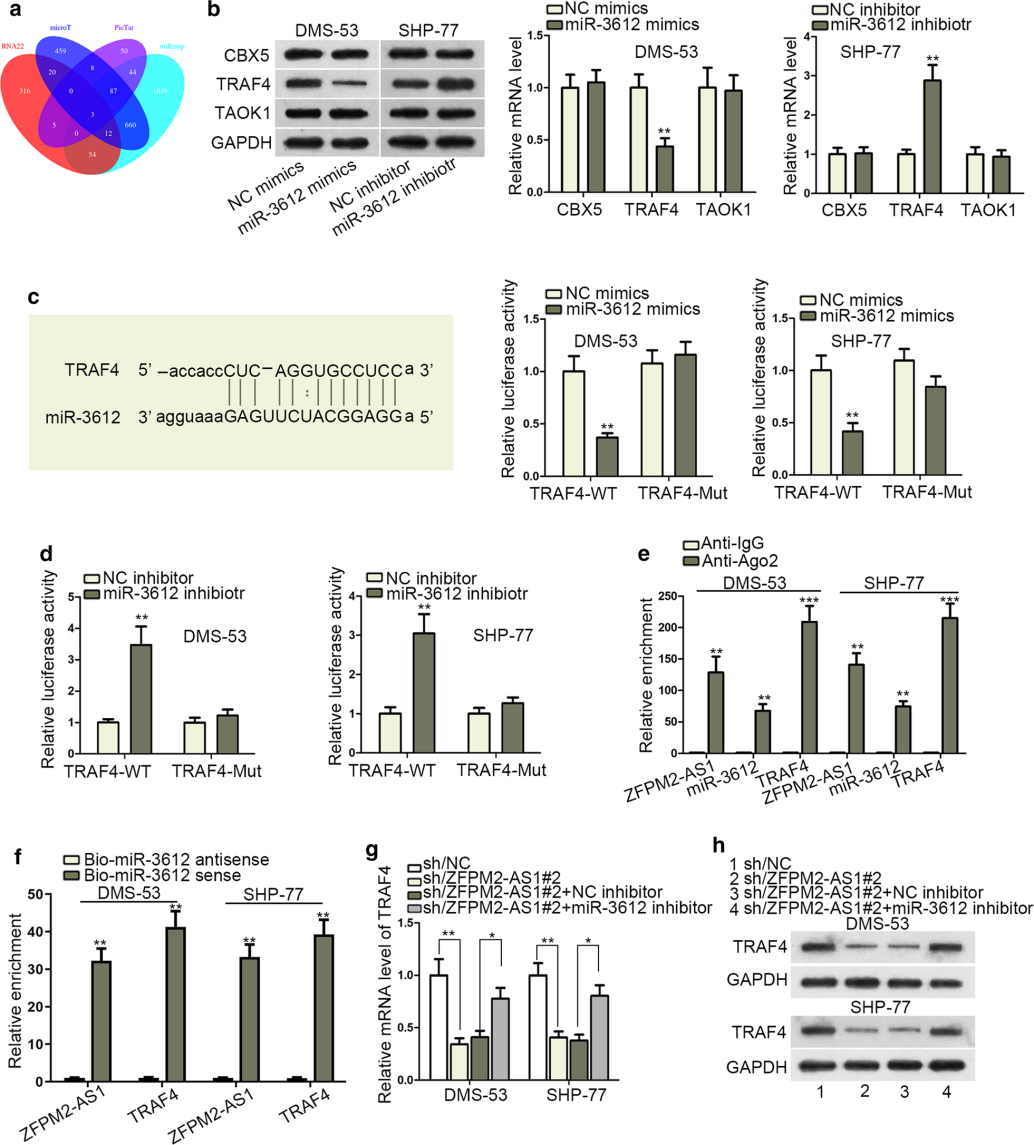
ZFPM2-AS1 upregulated TRAF4 expression via sequestering miR-3612. **a** Three downstream genes (TRAF4, CBX5, TAOK1) were predicted as potential targets of miR-3612. **b** qRT-PCR and western blot assays measured the mRNA and protein levels of TRAF4, CBX5 and TAOK1 in SHP-77 and DMS-53 cells. **c**, **d** Luciferase activity of TRAF4-WT or TRAF4-MUT was examined by luciferase reporter assay in SHP-77 and DMS-53 cells. **e** RIP assay tested the enrichment of TRAF4, miR-3612 and ZFPM2-AS1 in Anti-Ago2 group. **f** RNA pull down assay measured the enrichment of TRAF4 and ZFPM2-AS1 in Bio-miR-3612 sense group. **g**, **h** The mRNA and protein expressions of TRAF4 in differently transfected groups were assessed by qRT-PCR and western blot. *P < 0.05, **P < 0.01, *** P < 0.001

The decisive role of mRNAs in the regulating cancer progression induced us to find out the target gene of miR-3612. RNA22, microT, PicTar, and miRmap were employed for the exploration of miR-3612 targets. Three genes (CBX5, TRAF4, TAOK1) were revealed as candidates (Fig. 4a). Via the detections of qRT-PCR and western blot, miR-3612 negatively regulated TRAF4 expression in SHP-77 and DMS-53 cells (Fig. 4b). Additionally, TRAF4 was highly expressed in SCLC tissues rather normal tissues (Additional file 1: Fig. S1E). In a similar way, the predicted binding site of TRAF4 and miR-3612 was obtained. Luciferase reporter assay was conducted to evaluate the effectiveness of the indicated binding site. The activity of TRAF4-WT in SHP-77 and DMS-53 cells was repressed by miR-3612 mimics or simulated by its inhibitor (Fig. 4c, d). Besides, RIP assay demonstrated the co-existence of ZFPM2-AS1, miR-3612 and TRAF4 in Ago2-specific complex (Fig. 4e). Also, the enrichment of ZFPM2-AS1 and TRAF4 was detected in Bio-miR-3612 sense group (Fig. 4f). Subsequently, it verified that silencing of ZFPM2-AS1 decreased TRAF4 mRNA and protein expressions, while suppressing miR-3612 could countervail this repressing effect (Fig. 4g, h). It concluded that ZFPM2-AS1 competitively bound miR-3612 to upregulate TRAF4 expression in SCLC cells.

### ZFPM2-AS1 contributed to cell proliferation, invasion, migration and tumor growth in SCLC via upregulating TRAF4

**Fig. 5:**
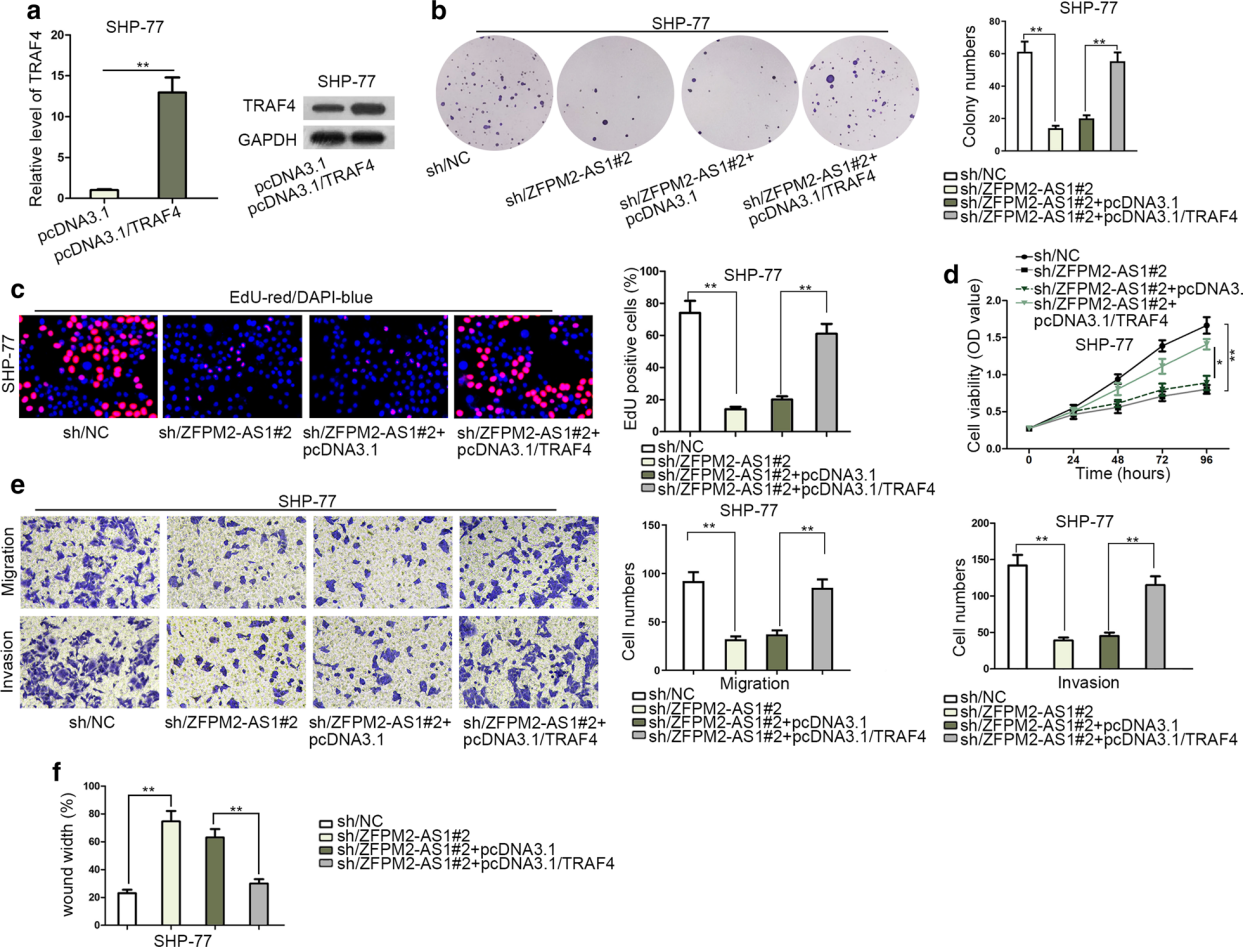
ZFPM2-AS1 contributed to SCLC proliferation, invasion, migration and tumor growth via upregulating TRAF4. **a** TRAF4 mRNA and protein expressions in SHP-77 cells were measured by qRT-PCR and western blot. **b**, **c** Colony formation and EdU analysis of SHP-77 cell proliferation following indicated transfection. In EdU assay, EdU-positive cells (stained in red) are cells that were proliferating, and DAPI stained cells (stained in blue) were total cells. **d** Transfected SHP-77 cell viability was analyzed by CCK-8. **e** Transwell assay of SHP-77 cell invasion and migration under indicated treatment conditions. **f** Wound healing assays of SHP-77 cell migration after indicated transfections. *P < 0.05, **P < 0.01

To certify whether TRAF4 affected the modulation function of ZFPM2-AS1 in SCLC cell growth, several rescue experiments were carried out. TRAF4 was overexpressed firstly, and its mRNA and protein levels were enhanced remarkably (Fig. 5a). Afterwards, TRAF4 elevation abolished the hindering effects of ZFPM2-AS1 depletion on cell proliferation (Fig. 5b–d). ZFPM2-AS1 inhibition induced the suppressed cell migration and invasion, while upregulating TRAF4 expression counteracted this inhibitory influence (Fig. 5e). Wound healing assay revealed that overexpression of TRAF4 attenuated the suppressive role of ZFPM2-AS1 deficiency on cell migration (Fig. 5f, Additional file 2: Figure S2A). On the other hand, in vivo experiments were also performed. The tumors extracted from mice injected with sh/ZFPM2-AS1#2-transfected cells were smaller and lighter than NC group. Then TRAF4 overexpression offset the suppressing effects of ZFPM2-AS1 depletion on tumor size, volume and weight (Additional file 2: Fig. S2B–D). In conclusion, ZFPM2-AS1 promoted SCLC via upregulating TRAF4 both in vitro and in vivo.

## Discussion

Recently, the biological importance of multiple lncRNAs in tumor progression has been emphasized extensively, indicating the possibility of the molecular-targeted application in treatment of tumors including SCLC. And the key role of lncRNAs in SCLC progression has been reported. For example, lncRNA HOTAIR is associated with cellular proliferation, invasion, and clinical reoccurrence in SCLC [25]. LncRNA TUG1 affects SCLC cell growth via regulating LIMK2b through EZH2 [26]. LncRNA PVT1 overexpression serves as a poor prognostic maker in SCLC, and facilitates malignant migration and invasion of cells [27]. In our research, lncRNA ZFPM2-AS1, located in 8q23.1, was an oncogenic gene in gastric cancer, renal cell cancer and lung adenocarcinoma [20, 22, 23]. Nonetheless, the role of ZFPM2-AS1 in SCLC was rarely discussed. In our research, we found its significantly high expression tendency in SCLC tissues and cell lines. Functionally, ZFPM2-AS1 insufficiency inhibited SCLC cell proliferation, invasion and migration in vitro and tumor growth in vivo, suggesting the carcinogenic role of ZFPM2-AS1 in SCLC.

Accumulating literatures showed that lncRNAs transcription could be attributed to specific transcriptional factors, including YY1. For instance, c-Myc activates lncRNA CCAT1 expression in gastric cancer [28]. STAT3 regulates the upregulation of lncRNA to affect in liver cancer metastasis [29]. YY1 contributes to lncRNA-PVT1 activation during lung cancer progression [30]. Besides, YY1, a ubiquitous protein in normal and tumor tissues, is identified as a potential prognosis biomarker and therapeutic target [31]. In this study, YY1 could bind with ZFPM2-AS1 promoter and transcriptionally activated ZFPM2-AS1 in SCLC cells.

After comprehending upstream regulation mechanism of ZFPM2-AS1, the downstream regulation mechanism also deserved to be discussed. Cytoplasmic lncRNAs participate in the ceRNA pattern in tumors. For illustration, lncRNA HOTAIR promotes HER2 expression through sponging miR-331-3p in gastric cancer [32]. LncRNA Unigene56159 accelerates EMT process in hepatocellular carcinoma via sponging miR-140-5p to regulate Slug [33]. Concerning the mechanism underlying ZFPM2-AS1 in SCLC, we hypothesized the ceRNA role of ZFPM2-AS1 in SCLC due to its large portion in SCLC cytoplasm. As expected, ZFPM2-AS1 could sponge miR-3612 to release TRAF4 expression. Moreover, the tumor-facilitator role of TRAF4 has been validated in hepatocellular carcinoma [34], intrahepatic cholangiocarcinoma [35], breast cancer [36], and colon cancer [37]. More importantly, TRAF4 is confirmed to promote the development of lung cancer [38–40]. And our study first illuminated that TRAF4 was involved in SCLC progression. TRAF4 overexpression reversed the suppressive function of ZFPM2-AS1 depletion on SCLC cell proliferation, invasion and migration in vitro as well as tumor growth in vivo.

## Conclusion

YY1-activated ZFPM2-AS1 promoted the malignant phenotypes of SCLC via sequestering miR-3612 to upregulate TRAF4, which possibly provide a promising candidate target for SCLC treatment.

## Supplementary information

**Additional file 1: Figure S1.** (A) The expression of ZFPM2-AS1 in SCLC tissues and matched normal tissues was detected by qRT-PCR. (B) The original picture of wound healing assay in Fig. 1g. (C–E) qRT-PCR measured the expression of YY1, miR-3612 and TRAF4 in SCLC tissues and matched normal tissues. **P < 0.01

**Additional file 2: Figure S2.** (A) The original picture of wound healing assay in Fig. 5f. (B-D) The pictures of tumors obtained from mice injected with differently transfected cells were taken. Tumor volume and weight were also measured. **P < 0.01

## Data Availability

Not applicable.

## Abbreviations

AFAP1-AS1: Actin filament associated protein 1 antisense RNA 1
ANOVA: Analysis of variance
ATCC: American Type Culture Collection
CCAT1: Colon cancer associated transcript 1
CCK-8: Cell counting kit-8
ceRNA: Competing endogenous RNA
ChIP: Chromatin immunoprecipitation
DMEM: Dulbecco’s modified eagle medium
EdU: 5-ethynyl-2-deoxyuridine
EZH2: Enhancer of zeste 2 polycomb repressive complex 2 subunit
FBS: Fetal bovine serum
FISH: Fluorescence in situ hybridization
HER2: erb-b2 receptor tyrosine kinase 2
HOTAIR: HOX transcript antisense RNA
IGF1R: Growth factor 1 receptor
LIMK2b: LIM domain kinase 2
LINC00488: Long intergenic non-protein coding RNA 488
LINC00511: Long intergenic non-protein coding RNA 511
lncRNAs: Long non-coding ncRNAs
MicroRNA: miRNA
MUT: Mutant
ncRNAs: Non-coding RNAs
NSCLC: Non-small‐cell lung cancer
PVDF: Polyvinylidene fluoride
PVT1: Pvt1 oncogene
qRT-PCR: Quantitative real-time polymerase chain reaction
RIP: RNA immunoprecipitation
RP4: RHO rhodopsin
SCLC: Small cell lung cancer
SDS-PAGE: Sodium dodecyl sulfate polyacrylamide gel electrophoresis
SH3GLB1: SH3 domain containing GRB2 like, endophilin B1
shRNAs: Short hairpin RNAs
SPF: Specific pathogen free
STAT3: Signal transducer and activator of transcription 3
TLN1: Talin 1
TRAF4: TNF receptor associated factor 4
TUG1: Taurine up-regulated 1
VMA21: Vacuolar ATPase assembly factor VMA21
WT: Wild
YY1: Yin Yang 1
ZFPM2-AS1: Zinc finger protein, FOG family member 2 antisense RNA 1

## References

[1] Bray F, Ferlay J, Soerjomataram I, Siegel RL, Torre LA, Jemal A (2018). Global cancer statistics 2018: GLOBOCAN estimates of incidence and mortality worldwide for 36 cancers in 185 countries. CA..

[2] Chen W, Zheng R, Baade PD, Zhang S, Zeng H, Bray F, Jemal A, Yu XQ, He J (2016). Cancer statistics in China, 2015. CA..

[3] Oronsky B, Reid TR, Oronsky A, Carter CA (2017). What’s New in SCLC? A Review. Neoplasia (New York, NY).

[4] Hann CL, Rudin CM (2008). Management of small-cell lung cancer: incremental changes but hope for the future. Oncology (Williston Park, NY).

[5] Tsoukalas N, Aravantinou-Fatorou E, Baxevanos P, Tolia M, Tsapakidis K, Galanopoulos M, Liontos M, Kyrgias G (2018). Advanced small cell lung cancer (SCLC): new challenges and new expectations. Ann Transl Med.

[6] Herrmann MK, Bloch E, Overbeck T, Koerber W, Wolff HA, Hille A, Vorwerk H, Hess CF, Muller M, Christiansen H (2011). Mediastinal radiotherapy after multidrug chemotherapy and prophylactic cranial irradiation in patients with SCLC–treatment results after long-term follow-up and literature overview. Cancer Radiother.

[7] Bunn PA, Minna JD, Augustyn A, Gazdar AF, Ouadah Y, Krasnow MA, Berns A, Brambilla E, Rekhtman N, Massion PP (2016). Small cell lung cancer: can recent advances in biology and molecular biology be translated into improved outcomes?. J Thor Oncol.

[8] Wang KC, Yang YW, Liu B, Sanyal A, Corces-Zimmerman R, Chen Y, Lajoie BR, Protacio A, Flynn RA, Gupta RA (2011). A long noncoding RNA maintains active chromatin to coordinate homeotic gene expression. Nature.

[9] Gomes AQ, Nolasco S, Soares H (2013). Non-coding RNAs: multi-tasking molecules in the cell. Int J Mol Sci.

[10] Liang H, Zhang J, Zen K, Zhang CY, Chen X (2013). Nuclear microRNAs and their unconventional role in regulating non-coding RNAs. Protein Cell.

[11] Tay Y, Rinn J, Pandolfi PP (2014). The multilayered complexity of ceRNA crosstalk and competition. Nature.

[12] Rashid F, Shah A, Shan G (2016). Long non-coding RNAs in the cytoplasm. Genom Proteom Bioinform.

[13] Feng K, Liu Y, Xu LJ, Zhao LF, Jia CW, Xu MY (2018). Long noncoding RNA PVT1 enhances the viability and invasion of papillary thyroid carcinoma cells by functioning as ceRNA of microRNA-30a through mediating expression of insulin like growth factor 1 receptor. Biomed Pharmacother.

[14] Liu ML, Zhang Q, Yuan X, Jin L, Wang LL, Fang TT, Wang WB (2018). Long noncoding RNA RP4 functions as a competing endogenous RNA through miR-7-5p sponge activity in colorectal cancer. World J Gastroenterol.

[15] Gao J, Yin X, Yu X, Dai C, Zhou F (2019). Long noncoding RNA LINC00488 functions as a ceRNA to regulate hepatocellular carcinoma cell growth and angiogenesis through miR-330-5. Digest Liver Dis.

[16] Shi X, Sun M, Liu H, Yao Y, Song Y (2013). Long non-coding RNAs: a new frontier in the study of human diseases. Cancer Lett.

[17] Li TT, He RQ, Ma J, Li ZY, Hu XH, Chen G (2018). Long noncoding RNAs in small cell lung cancer: a potential opening to combat the disease (Review). Oncol Rep.

[18] Lian Y, Xiong F, Yang L, Bo H, Gong Z, Wang Y, Wei F, Tang Y, Li X, Liao Q (2018). Long noncoding RNA AFAP1-AS1 acts as a competing endogenous RNA of miR-423-5p to facilitate nasopharyngeal carcinoma metastasis through regulating the Rho/Rac pathway. J Exp Clin Cancer Res.

[19] Lu G, Li Y, Ma Y, Lu J, Chen Y, Jiang Q, Qin Q, Zhao L, Huang Q, Luo Z (2018). Long noncoding RNA LINC00511 contributes to breast cancer tumourigenesis and stemness by inducing the miR-185-3p/E2F1/Nanog axis. J Exp Clin Cancer Res.

[20] Kong F, Deng X, Kong X, Du Y, Li L, Zhu H, Wang Y, Xie D, Guha S, Li Z (2018). ZFPM2-AS1, a novel lncRNA, attenuates the p53 pathway and promotes gastric carcinogenesis by stabilizing MIF. Oncogene.

[21] Yan J, Zhou C, Guo K, Li Q, Wang Z (2019). A novel seven-lncRNA signature for prognosis prediction in hepatocellular carcinoma. J Cell Biochem.

[22] Liu JG, Wang HB, Wan G, Yang MZ, Jiang XJ, Yang JY (2019). Long noncoding RNA ZFPM2-AS1 promotes the tumorigenesis of renal cell cancer via targeting miR-137. Eur Rev Med Pharmacol Sci.

[23] Xue M, Tao W, Yu S, Yan Z, Peng Q, Jiang F, Gao X (2019). lncRNA ZFPM2-AS1 promotes proliferation via miR-18b-5p/VMA21 axis in lung adenocarcinoma. J Cell Biochem.

[24] Knauss JL, Miao N, Kim SN, Nie Y, Shi Y, Wu T, Pinto HB, Donohoe ME, Sun T (2018). Long noncoding RNA Sox2ot and transcription factor YY1 co-regulate the differentiation of cortical neural progenitors by repressing Sox2. Cell Death Dis.

[25] Ono H, Motoi N, Nagano H, Miyauchi E, Ushijima M, Matsuura M, Okumura S, Nishio M, Hirose T, Inase N (2014). Long noncoding RNA HOTAIR is relevant to cellular proliferation, invasiveness, and clinical relapse in small-cell lung cancer. Cancer Med.

[26] Niu Y, Ma F, Huang W, Fang S, Li M, Wei T, Guo L (2017). Long non-coding RNA TUG1 is involved in cell growth and chemoresistance of small cell lung cancer by regulating LIMK2b via EZH2. Mol Cancer.

[27] Huang C, Liu S, Wang H, Zhang Z, Yang Q, Gao F (2016). LncRNA PVT1 overexpression is a poor prognostic biomarker and regulates migration and invasion in small cell lung cancer. Am J Transl Res.

[28] Yang F, Xue X, Bi J, Zheng L, Zhi K, Gu Y, Fang G (2013). Long noncoding RNA CCAT1, which could be activated by c-Myc, promotes the progression of gastric carcinoma. J Cancer Res Clin Oncol.

[29] Wang H, Huo X, Yang XR, He J, Cheng L, Wang N, Deng X, Jin H, Wang N, Wang C (2017). STAT3-mediated upregulation of lncRNA HOXD-AS1 as a ceRNA facilitates liver cancer metastasis by regulating SOX4. Mol Cancer.

[30] Huang T, Wang G, Yang L, Peng B, Wen Y, Ding G, Wang Z (2017). Transcription factor YY1 modulates lung cancer progression by activating lncRNA-PVT1. DNA Cell Biol.

[31] Bonavida B, Kaufhold S (2015). Prognostic significance of YY1 protein expression and mRNA levels by bioinformatics analysis in human cancers: a therapeutic target. Pharmacol Ther.

[32] Liu XH, Sun M, Nie FQ, Ge YB, Zhang EB, Yin DD, Kong R, Xia R, Lu KH, Li JH (2014). Lnc RNA HOTAIR functions as a competing endogenous RNA to regulate HER2 expression by sponging miR-331-3p in gastric cancer. Mol Cancer.

[33] Lv J, Fan HX, Zhao XP, Lv P, Fan JY, Zhang Y, Liu M, Tang H (2016). Long non-coding RNA Unigene56159 promotes epithelial-mesenchymal transition by acting as a ceRNA of miR-140-5p in hepatocellular carcinoma cells. Cancer Lett.

[34] Liu K, Wu X, Zang X, Huang Z, Lin Z, Tan W, Wu X, Hu W, Li B, Zhang L (2017). TRAF4 regulates migration, invasion, and epithelial–mesenchymal transition via PI3K/AKT signaling in hepatocellular carcinoma. Oncol Res.

[35] Kang Q, Zou H, Zhou L, Liu LX, Cai JB, Xie N, Li WH, Zhang C, Shi WH, Wang LM (2018). Role of the overexpression of TRAF4 in predicting the prognosis of intrahepatic cholangiocarcinoma. Int J Oncol.

[36] Zhu L, Zhang S, Huan X, Mei Y, Yang H (2018). Down-regulation of TRAF4 targeting RSK4 inhibits proliferation, invasion and metastasis in breast cancer xenografts. Biochem Biophys Res Commun.

[37] Yang K, Wang F, Han JJ (2015). TRAF4 promotes the growth and invasion of colon cancer through the Wnt/beta-catenin pathway. Int J Clin Exp Pathol.

[38] Kim E, Kim W, Lee S, Chun J, Kang J, Park G, Han I, Yang HJ, Youn H, Youn B (2017). TRAF4 promotes lung cancer aggressiveness by modulating tumor microenvironment in normal fibroblasts. Sci Rep.

[39] Chen T, Gao F, Feng S, Yang T, Chen M (2015). MicroRNA-370 inhibits the progression of non-small cell lung cancer by downregulating oncogene TRAF4. Oncol Rep.

[40] Li W, Peng C, Lee MH, Lim D, Zhu F, Fu Y, Yang G, Sheng Y, Xiao L, Dong X (2013). TRAF4 is a critical molecule for Akt activation in lung cancer. Cancer Res.

