# Supplementary figures and images for: YY1-induced lncRNA ZFPM2-AS1 facilitates cell proliferation and invasion in small cell lung cancer via upregulating of TRAF4
Source: Cancer Cell Int. 2020 Apr 3;20:108. doi: 10.1186/s12935-020-1157-7 (PMC7126398; doi:10.1186/s12935-020-1157-7)

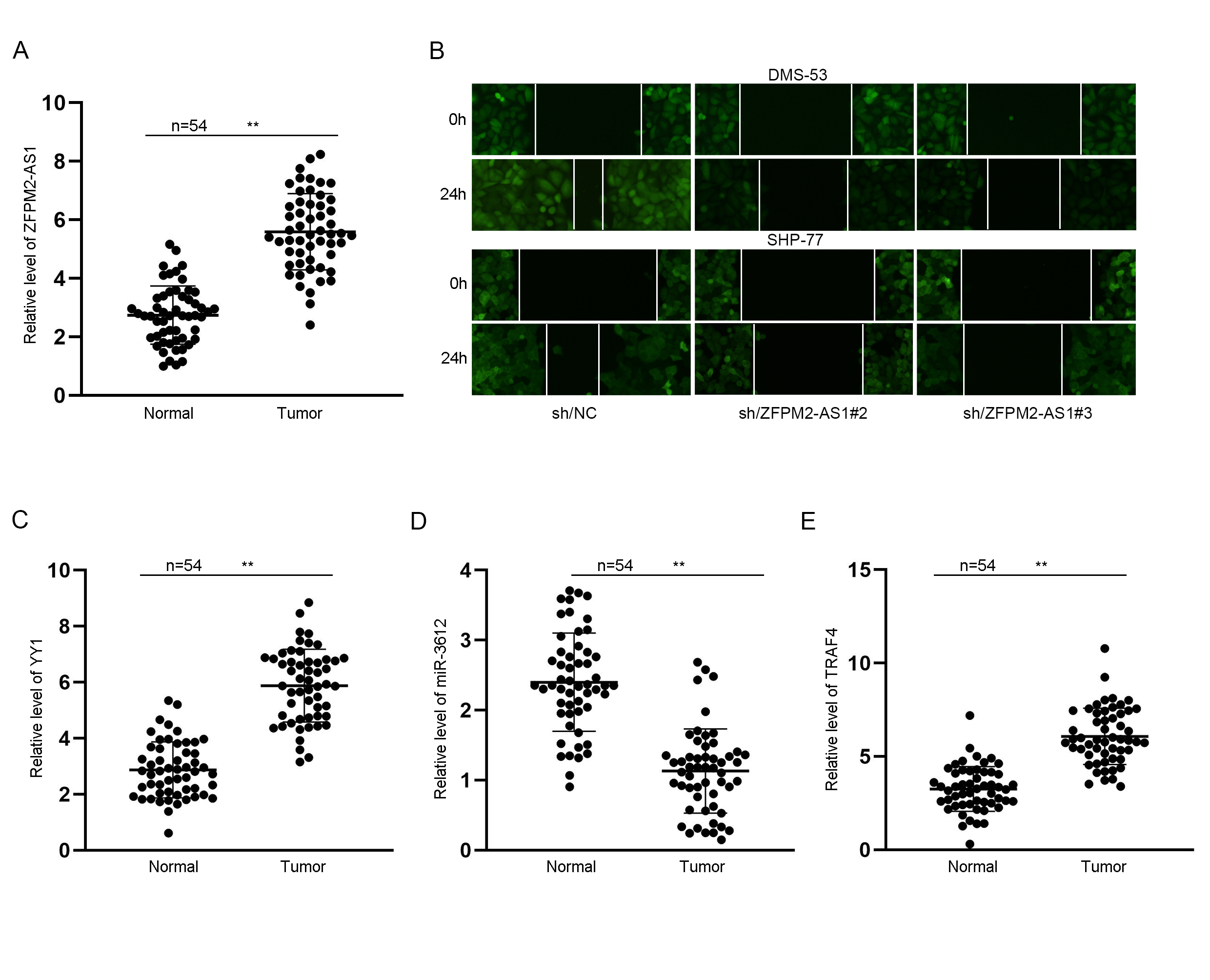

Supplement: Supplementary file 1 — Additional file 1: Figure S1. (A) The expression of ZFPM2-AS1 in SCLC tissues and matched normal tissues was detected by qRT-PCR. (B) The original picture of wound healing assay in Fig. 1g. (C–E) qRT-PCR measured the expression of YY1, miR-3612 and TRAF4 in SCLC tissues and matched normal tissues. **P < 0.01 [file 12935_2020_1157_MOESM1_ESM.tif]

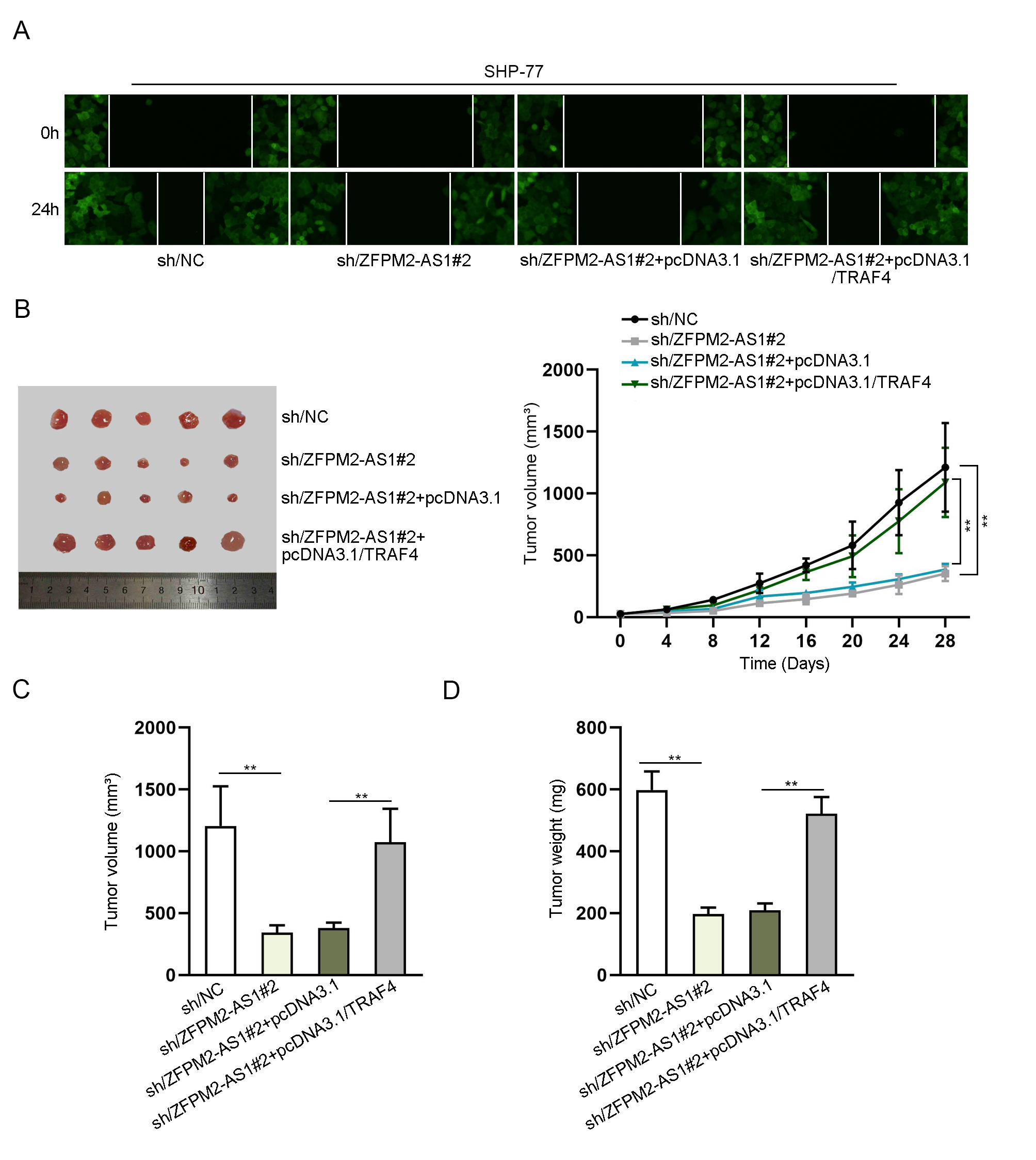

Supplement: Supplementary file 2 — Additional file 2: Figure S2. (A) The original picture of wound healing assay in Fig. 5f. (B-D) The pictures of tumors obtained from mice injected with differently transfected cells were taken. Tumor volume and weight were also measured. **P < 0.01 [file 12935_2020_1157_MOESM2_ESM.tif]
